# Medicinal Plants Used in Ethnoveterinary Practices in Adea Berga District, Oromia Region of Ethiopia

**DOI:** 10.1155/2021/5641479

**Published:** 2021-12-30

**Authors:** Megersa Feyisa, Addis Kassahun, Mirutse Giday

**Affiliations:** ^1^Faculty of Veterinary Medicine, Hawassa University, P.O. Box 05, Hawassa, Ethiopia; ^2^Aklilu Lemma Institute of Pathobiology, Addis Ababa University, P.O. Box 1176, Addis Ababa, Ethiopia

## Abstract

In Ethiopia, locally available materials, mainly medicinal plants, are commonly utilized to manage livestock diseases. However, this practice is currently being threatened by several factors including loss of traditional knowledge and depletion of plant resources. This calls for an urgent need to document the ethnoveterinary knowledge in the country and conserve the associated medicinal plants. The purpose of this study was, therefore, to document traditional knowledge on use of medicinal plants in the Adea Berga district, Oromia region of Ethiopia, to manage livestock ailments. Ethnobotanical data were collected largely through semistructured interviews conducted with purposively selected traditional healers of the district. The study identified 59 medicinal plants used in ethnoveterinary practices in the district. The great majority (90.4%) of the medicinal plants were used in fresh forms, which were mainly administered orally. The majority (65.4%) of the medicinal plants were gathered from the wild. Data revealed that yoke sore (wound) had the highest informant consensus factor (ICF) value (1.00), followed by leech infestation (0.92) and endoparasite infections (0.90). The highest fidelity level (FL) (100%) and rank order priority (ROP) (100%) values were obtained for the plants *Nicotiana tabacum*, *Malva parviflora*, and *Calpurnia aurea* that were used to treat leech infestation, retained placenta, and snake poisoning, respectively. Priority for further pharmacological and phytochemical investigations needs to be given to the aforementioned three plants with the highest FL and ROP values as such values may indicate their higher potency against the respective ailments.

## 1. Introduction

Ethiopia is one of the leading countries in the world and the first in Africa in terms of livestock population. However, livestock productivity is far below the possible expectations and, as a result, the country could not fulfill its local demands and hence imports animal products from other countries. One of the major challenges of livestock production in the country is the high prevalence of different diseases affecting domestic animals [1]. As most modern drugs are expensive and not affordable for the majority of Ethiopian farmers and pastoralists, disease control measures largely depend on traditional medicine, which mainly involves the use of medicinal plants [2]. According to estimate of Abebe and Ayehu [3], 95% of traditional medical preparations in Ethiopia are of plant origin.

However, despite the huge contribution of medicinal plants in the Ethiopian animal healthcare system and rich traditional medical knowledge, little effort has so far been made to properly document ethnoveterinary-related knowledge and conserve the associated medicinal plants in an effort to ensure their better and sustainable uses. As a result, the majority of the knowledge has been left undocumented being exposed to serious depletion due to acculturation and expansion of modern education. It is worth noting that in the Ethiopian traditional medical system, knowledge is handed down from generation to generation largely by word of mouth with little culture of documentation and as a result there is high probability that a portion of it could be lost in the process. The continuation of the practice is also negatively affected by the depletion of medicinal plants used in the system mainly due to agricultural expansion and deforestation. Therefore, there is an urgent need for conducting more ethnoveterinary surveys in different parts of the country to save the traditional knowledge and the associated medicinal plants from further loss.

Some ethnobotanical surveys have so far been conducted in the Oromia region of Ethiopia, including that in Borana [2], Bale Mountains National Park [4], Chiro district [5], the Dabo Hana district of Illubabor zone [6], four districts of Jimma zone [7], selected districts of East Wollega zone [8], Horro Gudurru district [9], Yabelo and Liben districts [10], the Melkabello district of East Hararghe zone [11], the Midakegn district of West Shewa zone [12], Eastern Shewa and Arsi zones [13], Kelem Wollega zone [14], the Dale Sadi district of West Hararghe zone [15], the Berbere district of Bale zone [16], Wolmera district [17], and Ambo district [18], with the purpose of documenting knowledge exclusively focused on medicinal plants used to control livestock ailments. However, to the knowledge of the authors, there is no study conducted in the Adea Berga district to document medicinal plants traditionally used to manage livestock ailments. The purpose of this study was, therefore, to document traditional knowledge on the use of medicinal plants by people in the Adea Berga district, West Shewa zone, the Oromia region of Ethiopia, to manage livestock ailments.

## 2. Materials and Methods

### 2.1. Description of Study District

Ethnoveterinary study to document medicinal plants used to manage livestock diseases was conducted in the Adea Berga district, West Shewa zone, the Oromia regional state of Ethiopia. The Adea Berga district is located between 9° 12′ and 9° 37′N latitude and 38° 17′ and 38° 36′E longitude [19] at 70 km west of Addis Ababa and 35 km northwest of the Holeta town. The district shares borders with the Walmera district in the south, Ejerie district in the southwest, Meta Robi district in the west, and the Muger River in the north and east. Enchni is the administrative town of Adea Berga district. The district has altitudes that range between 1371 and 3169 meters above sea level [20]. In the district, rainfall ranges between 800 and 1400 mm and temperature between 10°C and 29°C [19]. The district is divided into 34 administrative subdistricts (kebeles), the smallest administrate unit in Ethiopia.

Human population of the district is estimated to be 184,618, of which 92,277 are males and 92,341 are females (Adea Berga District Health Bureau, unpublished report, 2021). Mixed crop and livestock farming systems are the mode of agriculture in the study area. According to unpublished data from Adea Berga District Livestock and Fisheries Resources Office obtained in 2018, the district is estimated to have 165,317 heads of cattle, 56,800 sheep, 30,092 goats, 72,965 poultry, 14,208 donkeys, and 519 mules. Fascioliasis, pasteurellosis, foot-and-mouth disease (FMD), tick infestation, lice infestation, black leg, African horse sickness, lumpy skin diseases, sheep pox, Newcastle diseases, fowl cholera, and fowl pox are the commonly occurring diseases in the district (Adea Berga District Rural and Agricultural Development Office, unpublished report, 2011).

### 2.2. Selection of Study Sites and Informants

A reconnaissance survey was conducted in the Adea Berga district in November 2018 with the purpose of identifying study sites and identification of informants. Accordingly, nine subdistricts distributed in three agroecological zones of the district were identified for the study with the recommendation of elders and local authorities. These were Bishaan Diimoo, Kaaloo, Haroo Lemman, Oddoo Modjo, Iluu Kitabaa, Caancoo Birrattee, Adadaa Soddolbee, Iluu Coqorsaa, and Odaa Dalotaa. The elders and local authorities also assisted in the purposive sampling of 63 traditional healers from the nine subdistricts of the Adea Berga district to be involved in interviews.

### 2.3. Data Collection

Ethnoveterinary survey was conducted between November 2018 and March 2019, and data were mainly collected through semistructured interviews conducted with the selected traditional healers following the method of Martin [21]. The interview was based on a checklist of questions prepared in English and later translated to Afaan Oromoo, the local language in the study district. During interviews, data mainly regarding demographic characteristics of respondents, local name of medicinal plants employed in the practice, plant part used, remedy preparation methods, route of administration, disease treated, dosage regimen, remedy storing options, and habitat of medicinal plants used were gathered.

### 2.4. Collection, Preparation, and Identification of Plant Specimens

After every interview, walks were made with each informant to gather specimens of the claimed medicinal plants. The collected specimens were properly pressed and dried and later identified by their scientific name with the help of botanists at the Aklilu Lemma Institute of Pathobiology (ALIPB), Addis Abba University (AAU), and the National Herbarium (AAU), and vouchers were deposited in a mini-herbarium at ALIPB.

### 2.5. Data Analysis

Ethnoveterinary data were tabulated in Microsoft Excel spreadsheets and analyzed using SPSS version 20 software. The most useful information gathered on medicinal plants was summarized using descriptive statistical methods such as frequencies and percentages. The fidelity level (FL) value was computed for every medicinal plant reported by three or more informants, which is a measure of the degree of agreement among informants in the selection of a given medicinal plant to treat a given ailment, an indication of the possible level of efficacy. The fidelity level value was calculated using the formula FL =  *Ip* /*Iu* × 100, where *Ip* is the number of informants who reported the utilization of medicinal plants against a specific ailment and *Iu* is the total number of informants who mentioned the same plant against any ailment [22]. However, plants with similar FL values but known to different numbers of informants may vary in their healing potential. Thus, a correlation index known as relative popularity level (RPL) is additionally needed to be computed, and the rank order priority (ROP) value is determined by multiplying FL value by RPL value to differentiate the healing potential of plants of similar FL values [22, 23]. The RPL values range between 0 and 1. The plants are categorized into “popular” (RPL = 1) and “unpopular” (RPL < 1) groups. In this study, popular plants were those cited by more than half of the highest number of informants who cited a plant against an ailment, which is 27. Accordingly, medicinal plants cited by 14 or more informants were considered popular and were assigned with an RPL value of 1, whereas medicinal plants that were mentioned by less than 14 informants were considered unpopular and were assigned with RPL values less than 1 and were determined by dividing the total number of informants who mentioned the plant against a given ailment by 14.

Informant consensus factor (ICF) values were computed to determine the level of agreement of informants in the district on use of medicinal plants to treat a given ailment category. Informant consensus factor values may help in the selection of medicinal plants for phytochemical and pharmacological studies [24]. Informant consensus factor values were calculated using the formula ICF=(*nur* − *nt*)/(*nur* − 1), where *nur* is the number of use-reports for a particular use category and *nt* is the number of taxa used for a particular use category by all informants [25].

## 3. Results

### 3.1. Demographic Characteristics of Informants

Out of the total 63 informants who were identified for the interviews, 51 (80.95%) were males and 12 (19.05%) were females. The majority (53.73%) of the informants were illiterate (Table 1).

### 3.2. Medicinal Plants Reported and Ailments Treated

The study recorded a total of 59 medicinal plants (distributed in 35 families) used in the Adea Berga district to treat 35 livestock ailments, of which 55 were identified to a species level, three to a genus level, and only one to a family level (Table 2). The family Asteraceae had the highest number of medicinal plants accounting for six (10.17%) of the total medicinal plants, followed by Euphorbiaceae, Lamiaceae, and Solanaceae, each contributing four (6.78%) medicinal plants. The families Cucurbitaceae, Fabaceae, and Poaceae contributed three (5.09%) species each, whereas families Brassicaceae, Myrsinaceae, Rosaceae, and Rutaceae contained two (3.39%) medicinal plants each. Each of the rest 24 families was represented by single species. Of the total medicinal plants, 31(52.54%) were herbs, 18 (30.51%) were shrubs, and 10 (16.95%) were trees.

### 3.3. Medicinal Plant Parts Used and Methods of Preparation

**Figure 1:**
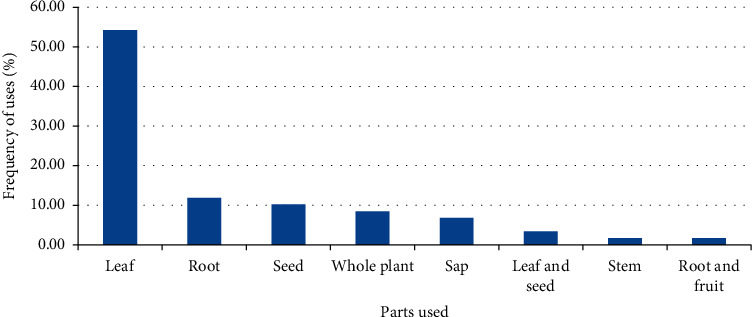
Parts of medicinal plants used in remedy preparation in the Adea Berga district.

Most remedies in the district were prepared from leaves accounting for 54.24% of the total medicinal plants reported, followed by plants that were harvested for their root (11.86%) and seeds (10.17%) (Figure 1). The most employed method of remedy preparation was crushing and squeezing or squeezing, accounting for 36 (61.02%) of the total preparations, followed by chewing, which accounted for 7 (11.86%) of the preparations. Seven (11.86%) medicinal plants are used in unprocessed form. Other methods of remedy preparation methods included grinding, chopping, roasting, and smoking.

### 3.4. Conditions of Medicinal Plants and Diluents Used in Remedy Preparations

Most of the medicinal plants used in remedy preparations were in their fresh form, accounting for 48 (81.36%) of the total claimed medicinal plants. Six (10.17%) medicinal plants were used either in dry or fresh form, and 5 (8.47%) were used in dry form. The commonly used diluent in the preparation of medicinal plant remedies was water accounting for 54.24% of the total preparations, followed by saliva (11%). Some (33.9%) of the medicinal plant did not need diluents in their preparations.

### 3.5. Routes of Remedy Administration

Nearly half (45.76) of the medicinal plants were administered through oral route, followed by those were administered topically (25.42) and nasally (10.17) (Table 3).

### 3.6. Informant Consensus Factor

Informant consensus factor (ICF) values for the different ailment categories were calculated, and the results show the highest score for yoke sore (wound) (1.00), followed by leech infestation (0.92), endoparasites infections (0.90), ectoparasites infections (0.88), retained placenta (0.88), snake poisoning (0.88), and evil eye (0.88) (Table 4). Medicinal plants used to treat ailment categories of high ICF values include *Aloe pubescens* for treating yoke sore; *Buddleja polystachya*, *Capsicum annuum*, *Millettia ferruginea*, and *Nicotiana tabacum* for treating leech infestation; and *Cucumis ficifolius*, *Embelia schimperi*, *Hagenia abyssinica*, and *Lepidium sativum* for treating endoparasites infections.

### 3.7. Fidelity Level and Rank Order Priority Values

Medicinal plants that scored the highest fidelity level (FL) (100%) and rank order priority (ROP) (100%) values include *Malva parviflora*, *Nicotiana tabacum*, and *Calpurnia aurea* that were used to expel retained placenta, remove leeches, and treat snake poisoning, respectively (Table 5).

### 3.8. Habitat of Medicinal Plants

**Figure 2:**
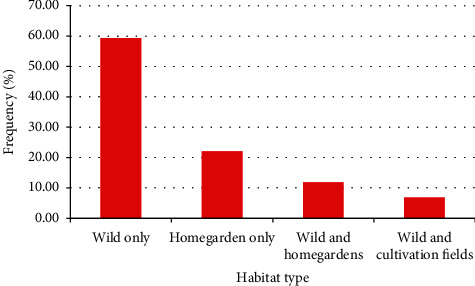
Proportions of medicinal plants harvested from different habitats in the Adea Berga district.

As revealed by the study, most (59.32%) of the medicinal plants used in remedy preparation in the study district were gathered from the wild only. Some (22.04%) were harvested from homegardens only, and other few were harvested from the wild or homegardens and from the wild or cultivation fields (Figure 2).

## 4. Discussion

### 4.1. Medicinal Plants Used and Ailments Treated

Livestock keeping is one of the most important economic sources of rural community in the Adea Berga district. Farmers in the district not only depend on plants to get fodder for their animals but also for their use as medicines to manage various livestock ailments as revealed by results of the current study. The study showed the use of 59 medicinal plant species distributed across 35 families to treat 35 livestock ailments. The highest number of medicinal plants used in remedy preparations belonged to the family Asteraceae, and this may be attributed to its dominance in terms of species diversity in the Ethiopian flora. Other studies conducted elsewhere in Ethiopia also reported the highest contribution of Asteraceae to the medicinal flora of the country [38, 47, 48]. The fact that herbs were the most frequently used growth forms in the preparation of remedies could be related to their relatively better abundance in the study district (as also witnessed by the investigators) and also due to their ease of preparation. Many other studies conducted elsewhere in the country also indicated the dominance of herbs in remedy preparations [8, 9, 12, 41, 49–52].

Of the 55 medicinal plants (identified to a species level) used in ethnoveterinary practices in the Adea Berga district, 26 were claimed to be used elsewhere in Ethiopia for other medical conditions not reported by the present study as indicated in Table 2 [2, 4–15, 17, 26–29, 31–40, 42–46], whereas 26 were claimed to be used elsewhere in the country at least once to manage same ailments [2, 5–12, 14, 15, 17, 18, 26–28, 30–34, 38–41, 44]. These include *Achyranthes aspera* (bleeding), *Aloe pubescens* (wound), *Brassica nigra* (bloat), *Brucea antidysenterica* (eye infection), *Buddleja polystachya* (leech infestation), *Calpurnia aurea* (snake bite and bloat), *Capsicum annuum* (leech infestation), *Croton macrostachyus* (bloat), *Dodonaea angustifolia* (wound and bone fracture), *Echinops kebericho* (evil eye), *Embelia schimperi* (endoparasites infections), *Eucalyptus globulus* (febrile illness), *Hagenia abyssinica* (endoparasites infections), *Hypericum revolutum* (diarrhea), *Justicia schimperiana* (coccidiosis), *Lepidium sativum* (bloat, colic, and endoparasites infections), *Linum usitatissimum* (retained placenta), *Nicotiana tabacum* (leech infestation and bloat), *Ocimum lamiifolium* (inappetence and febrile illness), *Olea europaea* subsp. *cuspidata* (snake poisoning), *Ricinus communis* (abscess), *Rubus steudneri* (diarrhea), *Ruta chalepensis* (bloat), *Verbascum sinaiticum* (blackleg and loss of appetite), *Vernonia amygdalina* (diarrhea), and *Zingiber officinale* (colic, bloat, and conjunctivitis) (see Table 2). Of these, the most frequently cited medicinal plants include *N. tabacum*, reported 16 times for its use to treat leech infestation [6, 8–10, 12, 14, 15, 17, 26, 28, 30–32, 39–41]; *C. macrostachyus*, reported 11 times for its use against bloat [2, 5, 7–9, 14, 32–34, 38, 39]; *H. abyssinica*, reported seven times for its use to treat endoparasites infections [11, 14, 15, 17, 28, 32, 40]; *L. sativum*, reported four times for its use to treat bloat [11, 12, 15, 34]; and *L. usitatissimum* reported four times for its use against retained placenta [6, 12, 15, 34]. High consensus among different communities in the use of a given medicinal plant to manage same ailment may indicate its better potency. According to Trotter and Logan [53], pharmacologically effective remedies are expected to have relatively greater informant consensus. *In vitro* studies conducted on *N. tabacum* [54, 55] revealed the killing effect of its leaf extracts against leeches, the effect of which is caused by an alkaloid called nicotine present in the plant [56, 57]. An *in vivo* experiment carried out on the crude extract of *H. abyssinica* demonstrated anthelmintic activity against cestodes in goats [58]. The substance kosotoxin present in *H. abyssinica* is believed to be responsible for the anthelmintic activity [59].

The study conducted in the Adea Berga district revealed for the first time the use of three medicinal plants in the Ethiopian ethnoveterinary practices. These include *Cynodon dactylon*, *Kalanchoe lanceolata*, and *Senecio ochrocarpus* that are used to treat snake poisoning, swelling of dewlap, and eye infection, respectively. Literature review revealed that so far, no study, exclusively conducted on medicinal plants of ethnoveterinary importance in the country, has reported the use of such plants.

### 4.2. Parts Used and Methods of Remedy Preparation and Routes of Administration

The fact that the leaf was the most commonly used medicinal plant part in remedy preparations in the district could be related to its better availability and ease of processing. It could also be due its richness in secondary metabolites [60]. The dominance of leaves in remedy preparations were also reported by studies conducted elsewhere in the country [61, 62].

The great majority of the remedies were claimed to be prepared from fresh plant materials, which might be attributed to the year-round availability of the same in the immediate environment. The better preference for fresh plant materials in the preparation of remedies was also reported by studies conducted elsewhere in the country [63]. A large proportion of the remedies in the study district were prepared by crushing or squeezing, which could be attributed to its ease of processing. Other studies [18, 31, 33, 40, 64] conducted elsewhere in the country also reported crushing as the commonly used method in the preparation of remedies. Availability and its property in dissolving many active compounds could be the reasons for the common use of water as diluent in the preparation remedies in the district as it is also the case in other parts of the country [18, 65, 66].

Oral is the most frequently employed route of remedy administration in the study district, and this could be related to its environment's suitability for rapid physiological reaction [7, 67]. Other studies conducted elsewhere in the country also reported the common use of oral route in administration of herbal preparations [4, 62].

### 4.3. Informant Consensus Factor, Fidelity Level, and Rank Order Priority Values

The fact that *Nicotiana tabacum* scored the highest FL and ROP values for its use to treat leech infestation, an ailment that scored the second highest ICF value, may indicate the high preference among informants in the study district for the plant to treat the ailment. Medicinal plants used to manage ailment categories of high ICF values and those of high FL and ROP values are expected to demonstrate good potency and thus are considered to be good candidates for further pharmacological and phytochemical investigations [22, 25]. *Nicotiana tabacum* is also used elsewhere in Ethiopia for treating leech infestation [6, 26]. *In vitro* investigation of leaf extracts of *Nicotiana tabacum* demonstrated activity against leeches [54, 55, 68], which could be attributed to the presence of nicotine in the leaf extract that interacts with nicotinic acetylcholine receptors to cause muscle weakness [55]. The other plant *Calpurnia aurea* that scored the highest FL and ROP values for its use to treat snake poisoning in the study district is also used elsewhere in the country against same ailments [27].

### 4.4. Comparison of Medicinal Plant Knowledge between Different Social Groups

Results of the present study indicate that the great majority of respondents selected for interviews in the study district were males. This may reflect the fact that males are the most favored by practitioners in the district in the transfer of traditional medical knowledge across generations. Other studies conducted elsewhere in the country also showed the dominance of males in traditional medical practices [7, 18, 69]. The majority of the participants selected for participation in ethnoveterinary study in the district were illiterate. This may reveal the fact that illiterate people are more knowledgeable as they are less exposed to cultural change compared with literate ones. Other studies carried out in other parts of the country also revealed that illiterate people held better traditional medical knowledge than literate ones [7, 27].

### 4.5. Habitats of Medicinal Plants

As shown by the study, the majority of the medicinal plants used in remedy preparations in the study district were harvested from the wild, an indication of the little practice of cultivating medicinal plants in the study district. The common use of plants harvested from the wild was also reported by other studies conducted elsewhere in the country [31, 47, 62, 70, 71].

## 5. Conclusion

The results of the present study revealed the high dependence of people in the Adea Berga district in the use of medicinal plants to manage livestock ailments, which could be due to the high prevalence of different diseases, limited access to modern veterinary care facilities, high prices of modern drugs, and diversity of local flora. Based on the demographic characteristic of people who participated in the study, knowledge on herbal medicine is mainly held by males and illiterate people. Priority for further pharmacological and phytochemical investigations needs to be given to the plants *Nicotiana tabacum*, *Malva parviflora*, and *Calpurnia aurea* that scored the highest FL and ROP values for their uses to treat leech infestation, retained placenta, and snake poisoning, respectively, as such values may indicate their higher potency against the respective ailments. Urgent action is needed to conserve *Aloe pubescens*, an Ethiopian endemic plant that has been included in the IUCN Red List as “near-threatened” species. *Aloe pubescens* is among the five medicinal plants in the study area that scored the highest FL value.

## Figures and Tables

**Table 1:** Demographic characteristics of informants in the Adea Berga district.

Parameters	Frequency	Percent (%)
Age, years		
25–40	20	31.75
40–45	32	50.79
Above 55	11	17.46
Sex		
Females	12	19.04
Males	51	80.95
Education level		
Illiterate	34	53.97
Elementary school	10	15.87
Secondary school	16	25.40
University degree holder	3	4.76

**Table 2:** Lists of medicinal plants used for the treatment of livestock diseases in the Adea Berga district.

Scientific name (family)	Local name	Habit	Parts used	Local (and English) names of disease treated	Animal treated	Mode of preparation	Administration route	Voucher number	Ethnoveterinary use elsewhere in Ethiopia
*Acacia abyssinica* Benth. (**Fabaceae**)	Laaftoo	Tree	Leaf	Biifaa (snake poisoning)	All domestic animals	Chewing	Nasal, oral	MF 040-2018	Goiter, stomachache [12]
*Achyranthes aspera* L. (**Amaranthaceae**)	Darguu	Herb	Leaf	Dhiiga dhaabuf (**bleeding**)	All domestic animals	Squeezing	Topical	MF 036-2018	Chronic trypanosomiasis, headache, babesiosis, gonorrhea, wound [26]; nasal infection, ophthalmic infection, **minor bleeding** [27]; mastitis, wound, diarrhea [28]; blackleg, mastitis, bleeding [8]; bloat [29]; blackleg [30]; wound, mastitis [9]; eye infection, anthrax, snake bite, wound, donkey's malaria [31]; abdominal discomfort, febrile disease [11]; blackleg [5]
*Ajuga integrifolia* Buch.-Ham. (**Lamiaceae**)	Armaa guusaa	Herb	Leaf	Michii (febrile illness)	All domestic animals	Crushing	Oral	MF 043-2018	Calf diarrhea [26]; internal parasite [32]; sprain [33]; diarrhea [17]
*Allium sativum* L. (**Alliaceae**)	Qullubii adii	Herb	Bulb	Madaa gogsuuf (wound)	All domestic animals	Crushing	Topical	-	Blackleg, dermatophilosis, mange, scabies, ringworm, parasitic leech, lice infestation in chicken, and helminthiasis [27]; mange and internal parasite [34]; hepatitis [4]; expectorant, antiseptic [2]; mastitis, colic, diarrhea, bloat, internal parasites, septicemia [6]; mastitis, colic diarrhea, bloat, internal parasites [15]; abdominal pain, blackleg, leech, pasteurellosis, bloat [8]; blackleg [7]; abdominal pain, pasteurellosis, bloat [9]; foot-and-mouth disease [14]; abdominal ache, bloat [14]; malaria and stomach problem [12]; mastitis, diarrhea, internal parasite [10]; chicken bloody diarrhea [35]; *Salmonella pullorum*, fowl cholera, coccidiosis, fowl typhoid, Newcastle disease [36]; cough, fungal infection, leech infestation [17]
*Aloe pubescens* Reynolds (**Aloaceae**)	Argiisa	Shrub	Sap	Madaa wanjoo (**yoke sore**)	Cattle	Squeezing	Topical	MF 017-2018	**Wound** [18]; trypanosomiasis, tsetse fly repellent [37]
*Artemisia abyssinica* Sch. Bip. ex A. Rich. (**Asteraceae**)	Arrittaa	Herb	Leaf	Budaa (evil eye)	All domestic animals	Unprocessed	Nasal	MF 029-2018	Epilepsy [4]
*Bersama abyssinica* Fresen. (**Melianthaceae**)	Lonchisaa	Tree	Leaf	Albaatii (diarrhea)	All domestic animals	Crushing and squeezing	Oral	MF 041-2018	Dermatophytes [4]; kidney problems, wound, swelling [38]; trypanosomiasis [6]; trypanosomiasis [15]; blackleg, paralyzed animal [14]; skin parasites [12]
*Brassica nigra* (L.) K. Koch (**Brassicaceae**)	Sanaficaa	Herb	Seed	Bokoka (bloat), rakkoo dakamuu nyaataa (simple indigestion), cininnaa (colic)	All domestic animals	Roasting	Oral	MF 003-2018	Bloat [14]
*Brucea antidysenterica* J.F. Mill. (**Simaroubaceae**)	Qomanyoo	Shrub	Leaf	Dhukkubbi ijaa (**eye infection**)	All domestic animals	Unprocessed	Ocular	-	Trypanosomiasis [6]; trypanosomosis [15]; rabies [8]; blackleg [7]; rabies, ring worms [9]; **blinded and eye discharged animal** [14]; epizootic lymphangitis [32]; malaria, rabies, toothache [12]; mastitis [10]; epizootic lymphangitis [39]
*Buddleja polystachya* Fresen. (**Buddlejaceae**)	Adaddoo	Shrub	Leaf	Hadhaandhula baasuf (**leech infestation**)	Cattle	Crushing and diluting	Nasal	MF 035-2018	**Leech infestation** [31]; eye disease [12]; internal parasite, diarrhea [39]
*Calpurnia aurea* (Aiton) Benth. (**Fabaceae**)	Ceekaa	Shrub	Leaf	Bofatu hiddee (**snake bite**), bokoka (**bloat**)	All domestic animals	Crushing and diluting	Nasal, oral	MF 024-2018	Tick infestation, helminthiasis, **snake bite**, sore and parasitic leech [27]; mite infestation, conjunctivitis [34]; dermatophytes [4]; skin disease, rabies [6]; mastitis, skin diseases like dermatophilosis and ectoparasites (lice, ticks) [28]; lice infestation, leech [8]; diarrhea, skin infection, blackleg, respiratory manifestations, ectoparasites [7]; cattle lice [30]; pasteurellosis, dermatophilosis, and ectoparasites [40]; lice infestation, leech infestation, abdominal pain, **bloat [9**]; ectoparasites, alopecia [14], lice infestation, ticks infestation [31]; mange [41]; internal parasites, pasteurellosis, mastitis, dermathophillosis, ectoparasites (tick, fleas, lice) [32]; scabies [12]; internal parasites [42]; flea and louse infestation [43]; internal and external parasite infection [10]; tsetse fly repellent [37]; *Estrus ovis*, trypanosomiasis [39]; rabies, external parasite [17]
*Capsicum annuum* L. (**Solanaceae**)	Barbaree	Herb	Seed	Hadhaandhula baasuf (**leech infestation**)	Cattle	Grinding and diluting	Nasal	-	Cowdriosis [2]; colic, bloat, septicemia [6]; abdominal pain, bloat, blackleg, pasteurellosis, **leech [8**]; pasteurellosis, **leech infestation**, tapeworm [9]; abdominal aches, **leech infestation** [14]; bloat, blackleg, colic [12]; chicken diarrhea and ectoparasite infection [35]; colic, bloat, internal parasites infection [17]
*Carissa spinarum* L. (**Apocynaceae**)	Agamsa	Shrub	Seed	Sirna fincanii (urinary tract infection)	All domestic animals	Grinding and diluting	Oral	MF 022-2018	Helminthiasis, parasitic leech [27]; evil eye, colic [34]; helminthiasis [2]; rabies [6]; ring worm, wound [8]; ring worm, wound [9]; febrile causal disease, evil eye [11]; evil eye, headache, stomachache, gonorrhea [12]
*Clausena anisata* (Willd.) Hook.f. ex Benth. (**Rutaceae**)	Ulummaa	Shrub	Leaf	Albaatii (diarrhea)	All domestic animals	Crushing and squeezing	Oral	MF 014-2018	Ectoparasite infestation [6]; lice [15]; blackleg, respiratory manifestations [7]; skin rash [12]
*Croton macrostachyus* Hochst. ex Delile (**Euphorbiaceae**)	Bakkanisa	Tree	Leaf	Bokoka (bloat), o'ichoo (foot rot)	Cattle, sheep, goat	Crushing and diluting	Oral, topical	MF 023-2018	Ringworm, dermatophilosis, mange, scabies, wound, minor bleeding, sore [27]; when a cow hates her calf, dermatophytosis, wound, bloat, eye defect [34]; anthelmintic [13]; equine colic, abdominal pain, bloat [38]; bloat [2]; mastitis, rabies, colic, trypanosomiasis, septicemia [6]; wound, fungal infection [15]; febrile illness in donkey (michii), expulsion uterine mass in camels [44]; ring worm, bloat, wound [8]; blackleg, trypanosomiasis, diarrhea, blackleg, helminthic infection, respiratory manifestations, bloat, lesion [7]; wound, fungal infection [40]; ring worm, bloat, wound [9]; bloat, dandruff [14]; equine colic, abdominal pain, bloat [32]; bloat, anthrax [33]; wound healing, antiparasitic [11]; ringworm, gonorrhea, scabies, evil eye, febrile illness, headache, wound, skin infection [12]; diarrhea (dysentery), external parasite [10]; chicken open wound [35]; blackleg, bloat [39]; blackleg, skin wound [17]; bloat, ringworm [5]
*Cucumis dipsaceus* Ehrenb. ex Spach (**Cucurbitaceae**)	Buqqee sexanaa	Herb	Sap	Maxxantuu alaa (ectoparasites infections), foroforii (scabies)	All domestic animals	Unprocessed	Topical (bathing)	MF 007-2018	Pneumonia, abdominal pain [26]; snake bite, rabies [40]; animal head parasites [33]; abdominal discomfort, bloat [11]; fowl cholera and coccidiosis [36]
*Cucumis ficifolius* A. Rich. **(Cucurbitaceae**)	Holotoo	Herb	Root, fruit	Dil'uu ture (retained placenta), rammoo garaa (endoparasites infections)	Cattle, sheep	Crushing and diluting	Oral	-	Coccidiosis, cowdriosis, hepatitis, wound [27]; blackleg [4]; blackleg, colic, emaciation [6]; rabies, anthrax [44]; blackleg [8]; rabies, trypanosomiasis, blackleg, cough [29]; blackleg [7]; rabies [9]; anthrax, abdominal pain [31]; febrile illness, ear pain, stomachache, cattle infection, tetanus, sudden sickness, inflammation [12]
*Cymbopogon citratus* (DC.) Stapf (**Poaceae**)	Xajjii saraa	Herb	Whole plant	Cininnaa (colic)	All domestic animals	Crushing and squeezing	Oral	MF 018-2018	Hepatitis, blackleg [4]
*Cynodon dactylon* (L.) Pers. (**Poaceae**)	Coqorsaa	Herb	Whole plant	Biifaa (snake poisoning)	All domestic animals	Chewing	Topical (spraying)	MF 009-2018	
*Datura stramonium* L. (**Solanaceae**)	Manjii	Herb	Leaf	Foroforii (ring worm)	All domestic animals	Crushing and squeezing	Topical (bathing)	MF 047-2018	Nerve problem [45]; yoke sore, wound, dermatophytosis, mastitis [34]; trypanosomiasis [29]; blackleg, respiratory manifestations [7]; anthrax [31]; blackleg, nasal bleeding [33]; wound [12]; coughing (for horses, mules, and donkeys) [43]; chicken depression and diarrhea [35]
*Dodonaea angustifolia* L. f. (**Sapindaceae**)	Ittachaa	Shrub	Leaf	Madaa gogsuuf (wound), caba (bone fracture)	All domestic animals	Crushing	Topical	MF 031-2018	Bloat, sudden diarrhea, ringworm, scabies [27]; saddle sore [34]; anthelmintic [13]; wound [28]; bloat, liver disease, diarrhea [7]; lice infestation [9]; dislocated bone [31]; bone dislocation [41]; retained placenta, dystocia [32]; ectoparasite [11]; wound, tapeworm [12]; blackleg [39]
*Echinops kebericho* Mesfin (**Asteraceae**)	Korobicho	Herb	Root	Budaa (**evil eye**)	All domestic animals	Smoking	Nasal	MF 045-2018	Coughing, pneumonia, pasteurellosis (mich), general illness, **evil eye**, taeniasis [34]; blackleg, cough [6]; diarrhea, blackleg [15]; blackleg, respiratory manifestations, liver disease, skin infection [7]; febrile illness [30]; diarrhea, blackleg [40]; dislocated bone [31]; anthrax [41]; blackleg, respiratory disease [11]; trypanosomiasis [37]
***Echinops* sp. (**Asteraceae)	Kosorruu	Shrub	Root	Dhukkubbi ijaa (ophthalmic disease)	All domestic animals	Chewing	Nasal	MF 044-2018	
*Embelia schimperi* Vatke (**Myrsinaceae**)	Hanquu	Shrub	Seed	Cininnaa (colic), maxxantuu keessaa (**endoparasites infections**)	All domestic animals	Roasting and crushing	Oral	MF 027-2018	Bloat, mucal diarrhea, bloody diarrhea [27]; anthelmintic [13]; **tapeworm** [12]
*Eucalyptus globulus* Labill. (**Myrtaceae**)	Bargamoo adii	Tree	Leaf	Kirkirsuu (shivering), loss of appetite, **michii** (febrile illness)	Cattle	Crushing and squeezing	Oral	MF 028-2018	**Mich**, lumpy skin disease [34]; ectoparasite [44]; blackleg [30]; avian cholera, influenza, skin disease, cough [12]; *Salmonella pullorum*, fowl cholera, coccidiosis, fowl typhoid, infectious coryza, Newcastle disease, fowl pox [36]
*Euphorbia* sp. **(Euphorbiaceae**)	Adamii	Shrub	Sap	Madaa gogsuuf (wound), kormammuu (wart)	All domestic animals	Crushing	Topical	MF 049-2018	
*Hagenia abyssinica* (Bruce ex Steud.) J.F.Gmel. (**Rosaceae**)	Heexoo	Tree	Leaf	Maxxantuu keessaa (endoparasites infections)	All domestic animals	Crushing And Diluting	Orally	MF 038-2018	Anthelmintic [13]; internal parasite, nasal bot [38]; **GIT parasite** [15]; **tape worm** in dogs [28]; **GIT parasite** [40]; **taeniasis** [14]; **internal parasite** [32]; **intestinal worms** [11]; fowl cholera, fowl typhoid and Newcastle disease [36]; **internal parasites infection** [17]
*Hypericum revolutum* Vahl (**Hypericaceae**)	Hindhee	Tree	Leaf	Albaatii (**diarrhea**)	Cattle, sheep	Crushing and squeezing	Oral	MF048-2018	Retained fetal membranes (RFM), metritis [40]; **calf diarrhea** [32]
*Juniperus procera* Hochst. ex Endl. (**Cupressaceae**)	Gattiraa	Tree	Leaf	Maxxantuu alaa (ectoparasites infections)	All domestic animals	Crushing and squeezing	Topical (bathing)	MF 005-2018	Trypanosomiasis [38]; retained placenta/fetal membrane [27]; diarrhea [4]; internal parasite, pasteurellosis [38]; abortion, irregular estrus [15]; abortion, irregular estrus, postpartum bleeding [40]; colic [17]
*Justicia schimperiana* (Hochst. ex Nees) T. Anderson (**Acanthaceae**)	Sansallii	Shrub	Leaf	Albaatii keelloo (**coccidiosis**)	Poultry	Crushing and squeezing	Oral	MF 002-2018	Wound [45]; diarrhea in hens, symptom of mouth, nose, and ocular discharge, blindness, depression, loss of appetence in hens [34]; rabies [6]; otitis [15]; jaundice, wound [28]; blackleg, rabies [8]; bloat, evil spirit [29]; diarrhea, blackleg, helminthic infection [7]; otitis, swollen of lymph node [40]; rabies, **coccidiosis** [9]; rabies [14]; dysentery [31]; rabies, blackleg, gonorrhea, malaria [12]; circling disease [39]; rabies, blackleg [17]
*Kalanchoe lanceolata* (Forssk.) Pers. (**Crassulaceae**)	Bosoqqee	Herb	Stem	Itoo maalaa (swelling of dewlap)	Cattle	Unprocessed	Implantation	MF 006-2018	
*Leonotis ocymifolia* (Burm.f.) Iwarsson (**Lamiaceae**)	Bokkolluu	Herb	Leaf	Albaatii (diarrhea), michii (febrile illness)	All domestic animals	Crushing and squeezing	Oral	MF 019-2018	Anthrax, blackleg [27]; anthrax [4]; blackleg [7]
*Lepidium sativum* L. (**Brassicaceae**)	Feexoo	Herb	Seed	Bokoka (**bloat**), cininnaa **(colic**), maxxantuu keessaa (**endoparasites infections**)	All domestic animals	Grinding and diluting	Oral	-	Blackleg, diarrhea, bloat [34]; blackleg, **colic**, diarrhea, bloat, **internal parasites**, septicemia [6]; blackleg, **bloat** [15]; blackleg [7]; diarrhea, skin infection [7]; blackleg [40]; cough [14]; swelling, anthrax [31]; blackleg, **colic**, diarrhea, **bloat, internal parasite** [11]; cough, **bloat**, malaria, diarrhea, tonsillitis, heart disease [12]; trypanosomiasis, tsetse fly repellent [37]; *Salmonella pullorum*, fowl cholera, coccidiosis, fowl typhoid, infectious coryza, Newcastle disease, fowl pox [36]
*Linum usitatissimum* L. (**Linaceae**)	Talbaa	Herb	Seed	Gogiinsa garaa (constipation), dhibee garaa (gastritis), dil'uu ture (retained placenta)	All domestic animals	Roasting, grinding and diluting	Oral	MF 020-2018	Placenta retention [34]; retained fetal membrane [6]; retained fetal membrane [15]; urine retention, coccidiosis [29]; retained placenta, dandruff [12]
*Maesa lanceolata* Forssk. (**Myrsinaceae**)	Abbayyii	Tree	Leaf and seed	Cittoo, madaa afaani (stomatitis)	Cattle	Crushing	Topical (painting)	MF 051-2018	Mange, tick infestation, dermatophilosis, helminthiasis, parasitic leech [27]; eye problem [4]; babesiosis, mastitis, kidney problem, internal parasite, wound, cancer (tumor) [38]; diarrhea, blackleg [15]; internal parasite [7]; diarrhea, blackleg [40]; leech infestation [32]; eye disease, pasteurellosis [12]; leech infestation [10]; leech infestation [10]; bloat [39]
*Malva parviflora* L. (**Malvaceae**)	Liitii	Herb	Root	Dil'uu ture (retained placenta)	Cattle, sheep	Crushing and squeezing	Oral	MF 013-2018	Wound, anthrax [28]
*Millettia ferruginea* (Hochst.) Baker (**Fabaceae**)	Birbirraa	Tree	Leaf	Hadhaandhula baasuf (leech infestation)	Cattle	Crushing and diluting	Nasal	MF 042-2018	Ectoparasite [34]; diarrhea [4]; tsetse fly repellent [37]; trypanosomiasis [39]
*Nicotiana tabacum* L. (**Solanaceae**)	Tamboo	Herb	Leaf	Hadhaandhula baasuf (**leech infestation**), bokoka (**bloat**)	Cattle, sheep	Crushing and diluting	Nasal, oral		Tick and **leech infestation**, snake bite, internal parasite, fever, wound infestation [26]; anthelmintic [13]; lice and some ectoparasite infestation, blackleg, trypanosomiasis, **leech infestation**, snake bite [6]; **leech infestation**, snake bite [15]; **leech** [35]; **leech** [8]; blackleg, respiratory manifestations, liver disease, snake bite, for fattening of cattle [7]; **leech infestation** [30]; **leech infestation** [40]; **leech infestation**, tapeworm [9]; cough, internal parasites [14]; **leech infestation**, scabies, lice infestation [31]; **leech infestation** [33]; **leech infestation**, tick infestation [32]; **bloat**, **leech infestation**, internal parasites, trypanosomiasis, eye infection, headache [12]; tick infestation [43]; **leech infestation** [10]; trypanosomiasis, tsetse fly repellent [37]; chicken depression [35]; trypanosomiasis, **leech infestation** [39]; snake poisoning, **leech infestation** [17]
*Ocimum lamiifolium* Hochst. ex Benth. (**Lamiaceae**)	Damakase	Shrub	Leaf	Kirkirsuu (shivering), qoqaa fuudhu (**inappetence), michii** (febrile illness)	Cattle	Crushing and squeezing	Oral	MF050-2018	Bloat, mucal diarrhea, **poor appetite**, bloody diarrhea [27]; pasteurellosis (**mich**) [34]; diarrhea [4]; blackleg [15]; abdominal colic, blackleg, trypanosomiasis, respiratory manifestations [7]; blackleg [40]; dingetengya (sudden sickness), blackleg [11]; diarrhea [10]; blackleg, diarrhea [17]
*Olea europaea* subsp. *Cuspidata* (Wall. & G.Don) Cif. (**Oleaceae**)	Ejersaa	Tree	Leaf	Biifaa (snake poisoning)	Cattle, sheep	Chewing	Topical (spraying)	MF 004-2018	Contagious caprine pleuropneumonia (CCPP) [46]; mange, ringworm, lumpy skin disease [27]; lumpy skin disease (LSD), gait problem [34]; pasteurellosis, calf diarrhea [38]; arthritis [15]; eye infection [7]; arthritis, paralysis, back bone pains [40]; rabies, **snake bite** [9]; dermatological disease [11]; hemorrhoid [12]; diarrhea [42]; blackleg, pneumonia, and bloat [10]; chicken depression [35]
*Olinia rochetiana* A.Juss. (**Oliniaceae**)	Dalachoo	Shrub	Leaf	Dhukkubii hir'isuf (pain)	All domestic animals	Chewing	Topical, oral	MF 033-2018	Calf diarrhea, wound [38]; mastitis, pneumonia, and other swellings or internal organs problems [10]
*Plantago lanceolata* L. (**Plantaginaceae**)	Qorxobbee	Herb	Leaf	Dhiiga dhaabuf (bleeding control)	All domestic animals	Squeezing	Topical	MF 016-2018	Rabies [27]; trypanosomiasis [29]; skin cut [12]
*Ranunculus multifidus* Forssk. (**Ranunculaceae**)	Marfataa	Herb	Root	Albaatii (diarrhea)	Sheep, cattle	Unprocessed	Tying	MF 026-2018	Dermatophilosis [27]; mastitis, internal parasite, trypanosomiasis [38]; trypanosomiasis [39]
*Rhamnus prinoides* L'Hér. (**Rhamnaceae**)	Geeshoo	Shrub	Leaf	Bokoka (bloat), hubaa qonqoo (tonsillitis)	Cattle, sheep	Crushing and squeezing	Oral	MF 025-2018	Abscess swelling [34]; nasal bot [38]; leech infestation [40]; equine colic, leech infestation, dandruff [32]; salmonellosis [11]; skin fungal infection, wound [12]; chicken depression [35]; fowl cholera, coccidiosis, fowl typhoid and Newcastle disease [36]; diarrhea, internal parasite [5]
*Ricinus communis* L. (**Euphorbiaceae**)	Hobo	Shrub	Leaf	Itoo (abscess), bu'aa (rectal prolapse)	All domestic animals	Chopping	Oral, topical	MF 021-2018	Blackleg, retained fetal membrane, skin disease [26]; mange, scabies, ringworm, retained placenta/fetal membrane [27]; diarrhea in new born, wound, sudden sickness, bloat, skin rashes/dermatitis [34]; retained fetal membrane [2]; rabies [6]; blackleg, actinomycosis [15]; retained fetal membrane, rabies [8]; blackleg, respiratory manifestations [7]; blackleg, actinomycosis [40]; retained fetal membrane, rabies [9]; wound [31]; anthrax, sudden sickness, bloat, actinomycosis, ulceric lymphangitis, epizootic lymphangitis [12]; mastitis [10]; foot and mouth disease (FMD) [17]; abscess, retained fetal membrane [5]
*Rubus steudneri* Schweinf. (**Rosaceae**)	Altufaa	Shrub	Leaf	Albaatii (**diarrhea**)	Cattle, sheep	Crushing and squeezing	Oral	MF 030-2018	Bloat**, mucal diarrhea**, **bloody diarrhea**, blackleg [27]; bloat**, diarrhea**, blackleg, constipation with mucoid feces [34]; swelling [29]
*Rumex nepalensis* Spreng. (**Polygonaceae**)	Shultii	Herb	Root	Cininnaa (colic)	All domestic animals	Crushing and squeezing	Oral	MF 012-2018	Internal parasite, headache [26]; bloody diarrhea [34]; blackleg [4]; fire burn [31] stomachache, spider poison, amoeba [12]; cough [39]; internal parasites infection, external parasites infection, wound [17]
*Ruta chalepensis* L. (**Rutaceae**)	Xeena adamii	Herb	Leaf, seed	Bokoka (**bloat**)	Cattle, sheep	Grinding	Oral	MF 011-2018	Retained placenta, abdominal pain, snake bite [26]; blackleg [4]; blackleg, anthrax [40]; poisoning [14]; equine colic, abdominal pain [32]; ear pain, evil eye, heart failure [11]; stomachache [12]; wound [42]; chicken welling of head [35]; coccidiosis, fowl typhoid & Newcastle disease [36]; **bloat** [17]; colic, pasteurellosis [5]
*Salvia nilotica* Juss. ex Jacq. (**Lamiaceae**)	Abbaa jarraa	Herb	Leaf	Albaatii (diarrhea)	All domestic animals	Crushing and squeezing	Oral	MF 015-2018	Blackleg [4]; mastitis, wound [38]
*Senecio ochrocarpus* Oliv. & Hiern (**Asteraceae**)	Monyoor	Herb	Leaf	Dhukkubii ijaa (eye infection)	All domestic animals	Chewing	Ocular	MF 032-2018	
*Solanum marginatum* L. f. (**Solanaceae**)	Hiddii	Shrub	Sap	Madaa dugdaa (back sore)	Horse	Squeezing	Topical	MF 034-2018	Dermatophytes [4]; cough [29]; blackleg, respiratory manifestations [7]; blackleg [14]; wound, breathing problem, anthrax [31]
*Stephania abyssinica* (Quart.-Dill. & A.Rich.) Walp. (**Menispermaceae**)	Kalalaa	Herb	Whole part	bokoka (bloat)	Cattle, sheep	Crushing and diluting	Oral	MF 037-2018	Contagious bovine pleuropneumonia (CBPP), calf pneumonia [27]; anthrax, rabies, foot, and mouth disease [34]; blocking/difficult in urination [41]; mastitis, blackleg, trypanosomiasis [39]; rabies [17]
*Tragia plukenetii* Radcl.-Sm. (**Euphorbiaceae**)	Dobbii	Herb	Root	Dil'uu ture (retained placenta)	Cattle, sheep	Crushing and diluting	Oral	-	Myiasis in goat [44]
*Verbascum sinaiticum* Benth. (**Scrophulariaceae**)	Gurraa harree	Herb	Leaf	Abbaa gorbaa (**blackleg**), fedhii nyaataf (**loss of appetite**)	Cattle	Crushing and diluting	Oral	MF 046-2018	Diarrhea, colic, snake bite, anthrax, wound, **blackleg**, myiasis, [34]; anthelmintic [13]; skin disease [6]; skin disease [15]; **blackleg**, swollen body, abdominal pain, **loss of appetite** [44]; wound [28]; thinning, rabies, trypanosomiasis, eye infection, mich [29]; cough [14]; wound, anthrax, dislocated bone [31]; anthrax [33]; febrile causal disease, heart disease, renal disease [11]; anthrax [17]
*Vernonia amygdalina* Delile (**Asteraceae**)	Eebicha	Shrub	Leaf	Albaatii (**diarrhea**)	cattle, sheep, goat	Crushing and squeezing	Oral	MF 001-2018	Trypanosomiasis, cough, wound, skin disease [26]; retained placenta/fetal membrane, CBPP [27]; colic, leech [34]; anthelmintic [13]; hepatitis [4]; lumpy skin disease, pasteurellosis, internal parasite [38]; GIT parasite, **diarrhea**, blackleg [15]; increase milk production [8]; **diarrhea**, blackleg, helminthic infection [7]; blackleg, respiratory manifestations, to improve milk production in cows, retained placenta [7]; GIT parasite, **diarrhea,** blackleg [40]; retained placenta [9]; wound [14]; equine colic, pasteurellosis, abdominal pain [32]; abdominal pain, mich, febrile disease [11]; internal parasites [42]; **diarrhea** and skin problem [10]; trypanosomiasis [37]; **chicken watery diarrhea** [35]; internal parasite, **diarrhea**, colic [39]; internal parasite, retained placenta [17]; **diarrhea**, internal parasites [5]
*Vernonia* sp. **(Asteraceae**)	Osolee	Herb	Leaf	Maxxantuu alaa (ectoparasites infections)	All domestic animals	Unprocessed	Topical (bathing)	MF 008-2018	
*Zehneria scabra* Sond. (**Cucurbitaceae**)	Hiddaa adii	Herb	Whole part	Martee (contagious bovine pleuropneumonia (CBPP))	Cattle	Unprocessed	Tying on the neck	MF 039-2018	Mich, yoke sore, dermatitis [34]; blackleg, pasteurellosis [8]; thinning [29]; rabies, pasteurellosis [9]; cough, emaciation, lameness, alopecia, colic [14]; trypanosomiasis [32]; swelling, rabies [12]; chicken swelling of head [35]
*Zingiber officinale* Roscoe (**Zingiberaceae**)	Zanjabilaa	Herb	Root	Cininnaa (**colic**), bokoka (**bloat)**, dhukubbii ijaa (**conjunctivitis**)	All domestic animals	Crushing	Oral	-	**Bloat,** bloody diarrhea, poor appetite, mucal diarrhea [27]; colic, eye defect, diarrhea due to stomach parasite [34]; **colic**, diarrhea, **bloat**, internal parasites [6]; **colic**, diarrhea, **bloat**, internal parasites [15]; blackleg, pasteurellosis, abdominal pain, leech [8]; **eye inflammation [7**]; rabies, pasteurellosis, abdominal pain, leech infestation [9]; ascariasis, stomach disorder [14]; contagious caprine pleuropneumonia (CCPP)/cough [42]; Newcastle diseases (fengile) [35]; coccidiosis, fowl typhoid and Newcastle disease [36]; **eye inflammation** [17]
**- (Poaceae**)	Hadduffaa	Herb	Whole part	Biifaa (snake poisoning)	All domestic animals	Chewing	Topical (spraying)	MF 010-2018	

*Note.* Disease names in bold indicate the use of the corresponding medicinal plant for the same purpose elsewhere in Ethiopia.

**Table 3:** Route of administration of medicinal plants in the Adea Berga district.

Route of administration	Frequency	Percent (%)
Oral	27	45.76
Topical	15	25.42
Nasal	6	10.17
Nasal and oral	3	5.08
Oral and topical	3	5.08
Ocular	2	3.39
Tying	2	3.39
Implantation	1	1.69

**Table 4:** Informant consensus factor values for the different ailment categories in the Adea Berga district.

Ailment category	Number of plant species	Number of use-reports	ICF value
Yoke sore (wound)	1	9	1.00
Leech infestation	4	41	0.92
Endoparasites infections	4	43	0.90
Ectoparasites infections	4	27	0.88
Retained placenta	4	27	0.88
Snake poisoning	5	35	0.88
Evil eye	2	10	0.88
Bleeding	2	8	0.85
Wound	4	18	0.82
Diarrhea	8	32	0.77
Bloat	9	35	0.76
Colic	7	22	0.71
Eye infection	4	11	0.70

**Table 5:** Rank order priority values of medicinal plants in the Adea Berga district scoring fidelity level values of 80% or more.

Plant name	Ailment category	*I*_U_	*I*_P_	FL value (100%)	RPL	ROP
*Malva parviflora*	Retained placenta	18	18	100.00	1.0	100.00
*Nicotiana tabacum*	Leech infestation	25	25	100.00	1.0	100.00
*Calpurnia aurea*	Snake poisoning	24	24	100.00	1.0	100.00
*Aloe pubescens*	Yoke sore	9	9	100.00	0.7	70.00
*Senecio ochrocarpus*	Eye infection	11	11	100.00	0.8	80.00
*Solanum marginatum*	Wound	15	13	86.66	1.0	86.66
*Cucumis dipsaceus*	Ectoparasites	27	24	88.89	1.0	88.89
*Vernonia amygdalina*	Diarrhea	20	17	85.00	1.0	85.00

*Note. I*_U_ = number of informants who mentioned the same plant against any ailment; *I*_P_ = number of informants who reported the utilization of medicinal plants against a specific ailment; FL = fidelity level; RPL = relative popularity level; ROP = rank order priority.

## Data Availability

Ethnoveterinary data were stored in a computer available at the Aklilu Lemma Institute of Pathobiology (ALIPB), Addis Ababa University (AAU), and the Faculty of Veterinary Medicine, Hawassa University. Readers may request the institutions for permission to get access to the data.
